# An Elaborate New Linker System Significantly Enhances the Efficacy of an HER2‐Antibody‐Drug Conjugate against Refractory HER2‐Positive Cancers

**DOI:** 10.1002/advs.202102414

**Published:** 2021-10-18

**Authors:** Seol Hwa Shin, Yun‐Hee Park, Seok Soon Park, Eun Jin Ju, Jin Park, Eun Jung Ko, Dong Jun Bae, Sang‐Yeob Kim, Chul‐Woong Chung, Ho Young Song, Se Jin Jang, Seong‐Yun Jeong, Si Yeol Song, Eun Kyung Choi

**Affiliations:** ^1^ Asan Medical Institute of Convergence Science and Technology Asan Medical Center University of Ulsan College of Medicine Seoul 05505 Republic of Korea; ^2^ Asan Institute for Life Sciences ASAN Medical Center Seoul 05505 Republic of Korea; ^3^ Asan Preclinical Evaluation Center for Cancer Therapeutics ASAN Medical Center Seoul 05505 Republic of Korea; ^4^ ADC R&D Center LegoChem Biosciences, Inc. Daejeon 34302 Republic of Korea; ^5^ Department of Convergence Medicine ASAN Medical Center University of Ulsan College of Medicine Seoul 05505 Republic of Korea; ^6^ Department of Pathology ASAN Medical Center University of Ulsan College of Medicine Seoul 05505 Republic of Korea; ^7^ Department of Radiation Oncology ASAN Medical Center University of Ulsan College of Medicine Seoul 05505 Republic of Korea

**Keywords:** antibody drug conjugate, HER2, patient derived xenograft (PDX)

## Abstract

Human epidermal growth factor receptor 2 (HER2) is overexpressed in breast and gastric cancers and this causes poor clinical outcomes. Although both T‐DM1 and Enhertu are approved as an HER2‐targeting antibody‐drug conjugate (ADC), the effects of these drugs are still not satisfactory to eradicate diverse tumors expressing HER2. To address this shortfall in HER2‐targeted therapeutics, an elaborate cleavable linker is created and a novel HER2‐targeting ADC composed with trastuzumab and monomethyl auristatin F, which is being investigated in a phase 1 clinical trial and is referred to as LegoChem Bisciences‐ADC (LCB‐ADC). LCB‐ADC displays a higher cytotoxic potency than T‐DM1 and it also has a higher G2/M arrest ratio. In animal studies, LCB‐ADC produces noticeable tumor growth inhibition compared with trastuzumab or T‐DM1 in an HER2 high‐expressing N87 xenograft tumor. Especially, LCB‐ADC shows good efficacy in terms of suppressing tumor growth in a patient‐derived xenograft (PDX) model of HER2‐positive gastric cancer as well as in T‐DM1‐resistant models such as HER2 low‐expressing HER2 low expressing JIMT‐1 xenograft tumor and PDX. Collectively, the results demonstrate that LCB‐ADC with the elaborate linker has a higher efficacy and greater biostability than its ADC counterparts and may successfully treat cancers that are nonresponsive to previous therapeutics.

## Graphical abstract

Antibody‐drug conjugate (ADC), delivering a severe toxin to selective tumor cells utilizing a high‐affinity of antibody has emerged as the most promising biotherapeutics for cancer. Here it is demonstrated that the application of the elaborate linker‐drug technology to human epidermal growth factor receptor 2‐ADC enables to eradicate the diverse cancer types rarely responding to previous therapeutics.

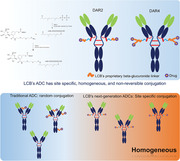

## 1 Introduction

Antibody‐drug conjugates (ADCs), the most notable anticancer therapeutics in terms of recent successes in the clinic, selectively deliver cytotoxic drugs to the desired tumor tissue.^[^ ^1^, ^2^ ^]^ Unlike chemotherapy for systemic treatment, ADC has the advantage of minimizing damage to normal cells while greatly enhancing the anticancer killing effect by targeting only cancer cells presenting the specific antigen. Because of this advantage, the development of ADCs has rapidly progressed; since Gemtuzumab ozogamicin was first approved by the US Food and Drug Administration (FDA) in 2000,^[^ ^3^ ^]^ more than 80 clinical trials are currently underway (www.clinicaltrials.gov). To date, nine ADCs targeting different cancer cell antigens with toxins conjugated by a linker have been approved by the FDA which are Gemtuzumab ozogamicin,^[^ ^4^ ^]^ brentuximab vedotin,^[^ ^5^ ^]^ trastuzumab emtansine,^[^ ^6^ ^]^ inotuzumab ozogamicin,^[^ ^7^ ^]^ polatuzumab vedotin‐piiq,^[^ ^8^ ^]^ enfortumab vedotin,^[^ ^9^ ^]^ trastuzumab deruxtecan,^[^ ^10^ ^]^ sacituzumab govitecan,^[^ ^11^ ^]^ and belantamab mafodotin^[^ ^12^ ^]^ for leukemia, lymphoma, myeloma, breast cancer, urothelial tumors, and other cancers. Without doubt, ADCs are now firmly established as the most promising targeted anticancer biotherapeutics.

Human epidermal growth factor receptor 2 (HER2) is encoded by the proto‐oncogene *ERBB2* and it is a plasma membrane bound protein harboring an extracellular domain. HER2 has shown an association with tumorigenesis and cancer progression and is a particularly important prognostic indicator of invasive breast cancer. Indeed, *ERBB2* gene amplification is observed in 20–25% of breast cancers and causes the overexpression of the HER2 protein. Moreover, HER2 is overexpressed and amplified in ≈20% of gastric cancer (GC) patients, where it is associated with a poor prognosis and is currently recognized as a new diagnostic factor and novel therapeutic target.^[^ ^14^, ^15^, ^16^ ^]^ Accordingly, HER2 is recognized as the first target for cancer therapy and assessing the HER2 status in GC tissue has become a routine approach to these lesions.^[^ ^15^ ^]^

A variety of HER2 targeting agents have been approved by the FDA in the United States, including trastuzumab, pertuzumab, lapatinib, neratinib, trastuzumab emtasine (trastuzumab‐DM1; T‐DM1, Kadcyla), and trastuzumab deruxtecan (fam‐trastuzumab deruxtecan‐nxki, Enhertu)^[^ ^13^ ^]^. Among these therapeutics, T‐DM1, an HER2‐targeting ADC (HER2‐ADC) that incorporates a thioether bond to the maytansanoid derivative emtasine, has been investigated for its possible clinical application in HER2‐positive metastatic breast cancer after neoadjuvant treatment with Herceptin and taxane chemotherapy.^[^ ^17^ ^]^ In the EMILIAstudy of trastuzumab emtansine versus capecitabine plus lapatinib in participants with HER2‐positive locally advanced or metastatic breast cancer, T‐DM1, compared to chemotherapy (lapatinib plus capecitabine), was shown to significantly improve the objective response rate (43.6% vs 30.8%), median overall survival (30.9 vs 25.1 month) and median progression‐free survival (9.6 vs 6.4 month) in HER2‐positive advanced breast cancer patients previously treated with trastuzumab and taxane.^[^ ^18^, ^19^ ^]^ Additionally, the KATHERINE study reported that the number of invasive disease‐free cases at 3 years was higher with T‐DM1 than with trastuzumab (88.3% vs 77.0%) and the risk of recurrence of invasive breast cancer or death was lower (by 50%) with adjuvant T‐DM1 than with trastuzumab for HER2‐positive early breast cancer patients with residual invasive disease after the completion of neoadjuvant therapy.^[^ ^20^, ^21^, ^22^ ^]^ Recently, Enhertu was approved in the United States for both unresectable and metastatic HER2‐positive breast cancers treated with two or more anti‐HER2 therapies.^[^ ^23^ ^]^ In the phase II DESTINY‐Breast01 trial, Enhertu was shown to have a durable antitumor effect with an overall response rate of 60.9% (complete response 6.0%, partial response (PR) 54.9%), a median progression‐free survival period of 16.4 months, and a median response duration of 14.8 months in previously treated HER2‐positive breast cancer patients.^[^ ^24^, ^25^ ^]^ Despite its established effectiveness in HER2‐positive breast cancer patients, several groups of patients, such as those with lower HER2 expression, are initially resistant or develop resistance to T‐DM1, and this drug was found to not to be superior to taxane in the GASTBYstudy of trastuzumab emtansine versus a taxane in previously treated HER2‐positive advanced gastric/gastroesophageal junction cancer.^[^ ^26^ ^]^ Hence, there is still an unmet need for effective targeting agents among low HER2‐expressing breast cancers and HER2‐positive gastric cancer patients and therapeutic options continue to be limited in these cases.

An ADC consists of three main structural units such as an antibody, cytotoxic agent and a linker, and all of them are important factors in the efficiency and safety of the ADC.^[^ ^27^, ^28^ ^]^ The suitability of using a platform technique to overcome the limitations previously observed for approved ADCs is now being investigated.^[^ ^29^ ^]^ The novel ADCs that have been proposed thus far in this regard are prepared by bonding a drug moiety with the lysine groups of an antibody, or by reducing all or part of the interchain disulfide groups of an antibody.^[^ ^30^ ^]^ These methods have some issues with regards to homogeneity, making quality control difficult.^[^ ^31^ ^]^ T‐DM1 is a nonreducible thioether heterogeneous lysine conjugate of trastuzumab with a drug‐to‐antibody ratio (DAR) of ≈3.5.^[^ ^17^ ^]^ Several latent weaknesses of T‐DM1 that have been reported include a finite Cmax due to toxicities, slow internalization rate, resistance mechanisms caused by a lack of intracellular trafficking and an increased expression of the drug transporters MDR1 and MRP1, and a deficiency in payload bystander effects.^[^ ^32^ ^]^ The ultimate goal of the linker is to allow an efficient drug release to the targeted cancer cells while maintaining a stable linkage between the antibody and the drug during circulation. Since the efficacy and systemic toxicity of the antibody‐drug conjugate thus depends on the stability of the linker, it plays a vital role in the safety of the ADC.^[^ ^33^ ^]^

We have developed an advanced linker‐drug technology platform that is universally applicable to manufacturing novel ADCs and was used to generate the most optimal HER2‐ADC. The advantages of our new linker technology include: i) an accurate DAR with high homogeneity achieved via site‐specific conjugation, due to prenylation of a CaaX body that can then be recognized by farnesyl transferase (FTase) at the end of the antibody; ii) efficient toxin release in cancer cells and increased linker stability in the bloodstream due to modified electron‐donating groups in a Seattle Genetics' beta‐glucuronide linker; and iii) a pharmacokinetic (PK) profile equivalent to that of an antibody.

We utilized our new ADC platform to produce a novel HER2‐ADC, which we refer to as an LCB‐ADC, and we verified its efficacy and suitability in various model systems where therapeutic limitations of ADCs had been described previously. Collectively, LCB‐ADCs using our cleavable linker exhibited a good mode of action, biostability, and effective anticancer efficacies in vitro and in vivo. We demonstrated in our present study that the application of our linker‐drug technology enables LCB‐ADCs to effectively target cancer cell types that were not responsive to previous therapeutics. LCB‐ADC1 is being investigated in phase 1, a dose‐escalation study in patients with HER2 expressed advanced solid tumors and local advanced or metastatic, HER2 positive breast cancer. This clinical trial was registered at clinicaltrials.gov (NCT03944499).

## 2 Results

### 2.1 LCB‐ADCs are Fabricated with a Novel Conjugation Method and Show an Improved Pharmacokinetic Profile

**Figure 1:**
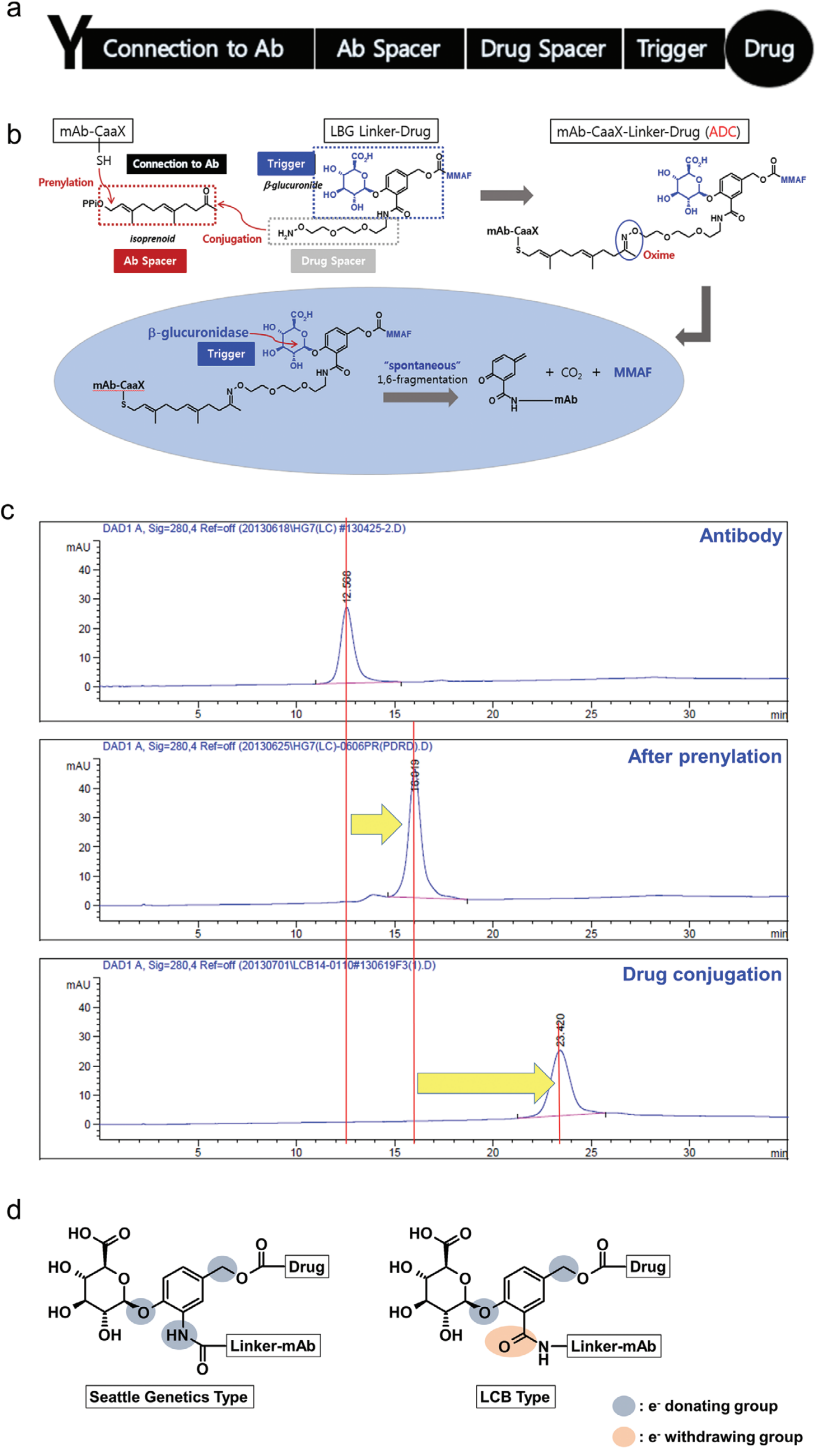
ADC conjugation strategy. a) Composition of the ADC. b) Conjugation process in vitro and drug release mechanism in cancer cells. c) Results of the hydrophobic interaction chromatography (HIC) analysis with the products in each step. d) Working hypothesis of the linker stability.

To achieve homogeneity of production and a smarter linker system for ADCs, a novel conjugation method was developed and an improved linker was designed (**Figure** **1**a,b). Additional amino acid sequences that can be recognized by farnesyl transferase (FTase) were added to the end of the antibody (light chain and/or heavy chain), termed the CaaX body. Bonding between the SH on the Cys residue of CaaX and the pyrophosphate of isoprenoid (farnesyl pyrophosphate) can then be generated by FTase (prenylation). An oxime bond is generated as a result of the subsequent reaction between the amine group of the linker‐toxin and the carbonyl (ketone) group of the prenylated CaaX body. The prenylation and drug‐conjugation step proceeded very efficiently with high homogeneity of the end product (Figure 1c and **Table** **1**). To increase the linker stability, the three electron‐donating groups in the Seattle Genetics beta‐glucuronide linker were modified. The electron density of the phenyl ring of the self‐immolative group, which is bonded to the *β*‐glucuronide ring, is relatively high and might be susceptible to metabolism (e.g., oxidation) and therefore is possibly less stable. Through a proprietary chemical modification, we prepared a reversed amide of the self‐immolative group (in relation to the directionality of the original amide), which became an electron‐withdrawing group. Its electron density is decreased and this stabilizes the linker (Figure 1d).

**Table 1:** Compositions of LCB‐ADC1, 2, and 3

ADC name	Linker type	Payload	DAR
LCB‐ADC1	PEG‐3	MMAF	2
LCB‐ADC2	PEG‐3,3,3	MMAF	4
LCB‐ADC3	PEG‐6,6,3	MMAF	4

**Table 2:** The PK parameters of Herceptin and LCB‐ADC1 in the rat and monkey

	Herceptin		LCB‐ADC1	
	Half‐life [day]	AUC^a)^ [µg × day mL^−1^]	Half‐life [day]	AUC^a)^ [µg × day mL^−1^]
Rat	13.4	689.3 ± 21.9	12.8	965.5 ± 33.5
Monkey	10.1	622.9 ± 26.5	8.6	684.8 ± 20.4

^a)^ Area under the curve.

**Figure 2:**
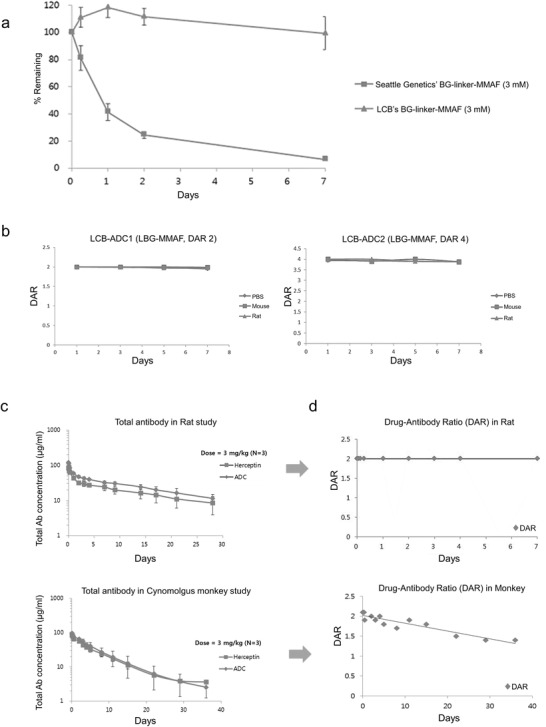
Stability of the linker‐toxin system and PK profiles of LCB‐ADC1. a) Comparison of linker‐drug stability in mouse plasma. b) Plasma stability of ADCs in mouse or rat plasma. c) PK profiles of Herceptin and LCB‐ADC1 in the rat and monkey. d) Drug‐antibody ratio (DAR) calculations.

We applied our mouse plasma stability tests to review our hypothesis and observed that the modified self immolative group (SIG)‐linker version is more stable in mouse plasma. This supports our hypothesis (**Figure** **2**a). The HER2‐targeting ADCs with the LCB type linker‐toxin (LCB‐ADC1 and LCB‐ADC‐2) maintain their stability for at least 7 d in mouse or rat plasma. When the PK profile was examined in the rat and monkey, the ADC was comparable to the original antibody (Herceptin). In addition, stable attachments of the linker toxin were observed in both species (Figure 2 and **Table** **2**).^[^ ^34^ ^]^ These results demonstrated that our novel HER2‐ADCs (LCB‐ADCs), that were elaboratively generated for a more precise DAR, show higher stability in mouse, rat and monkey serum than conventional ADCs.

### 2.2 A Potent Pharmacologic Activity of the LCB‐ADCs Is Induced by Monomethyl Auristatin F (MMAF)‐Mediated Tubulin Inhibition

**Figure 3:**
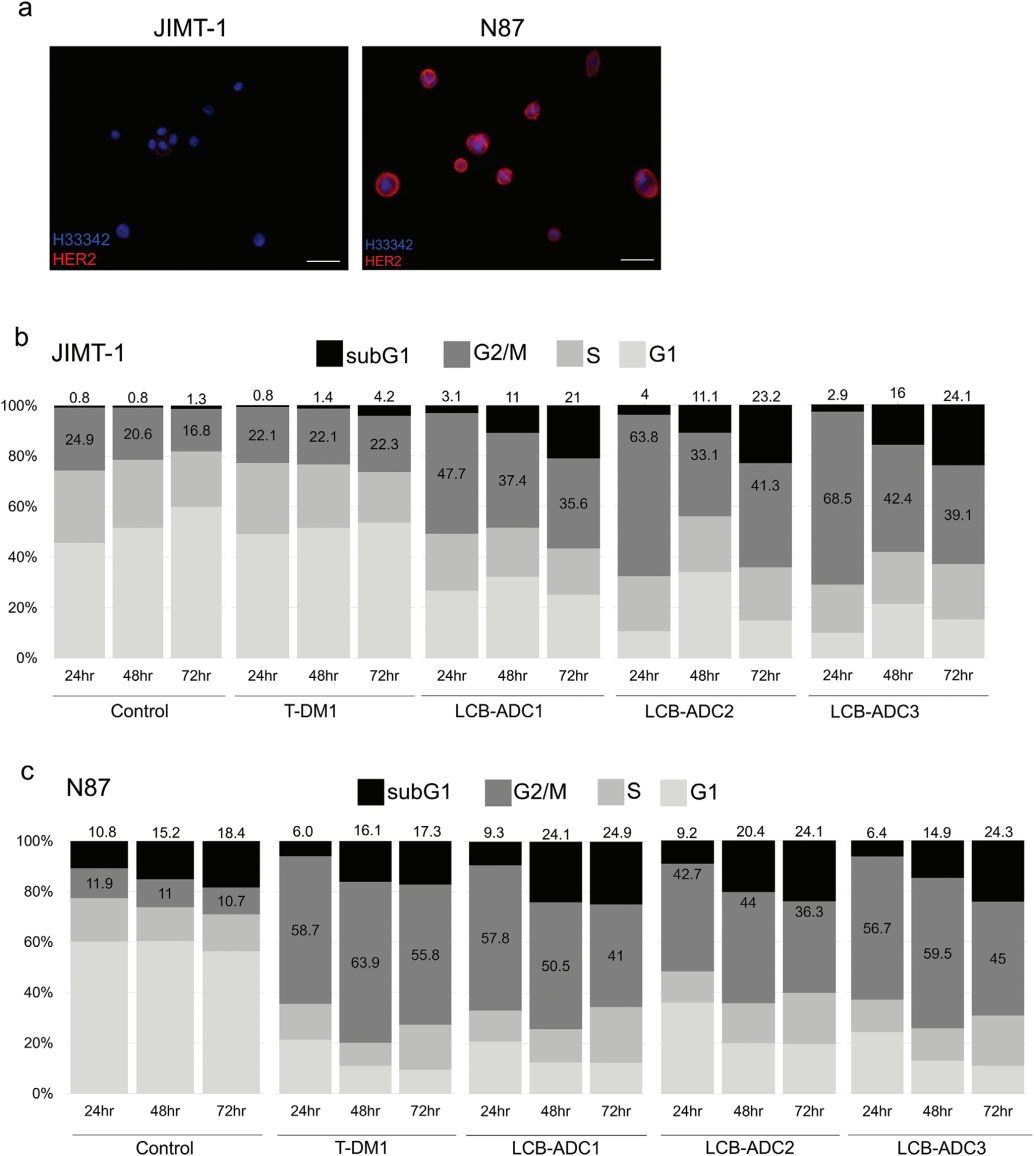
HER2 expression and cell cycle distribution in JIMT‐1 and N87 cells. a) HER2 expression in JIMT‐1 and N87 cells stained via ICC (red, Alexa 594‐stained HER2; blue, Hoechst‐stained nuclei, scale bar, 10 µm). Cell cycle distribution in b) JIMT‐1 and c) N87 cells treated with T‐DM1, LCB‐ADC1, or LCB‐ADC2 at 0.25 µg mL^−1^ for 72 h. Cells stained with propidium iodide (PI) were analyzed by flow cytometry.

**Figure 4:**
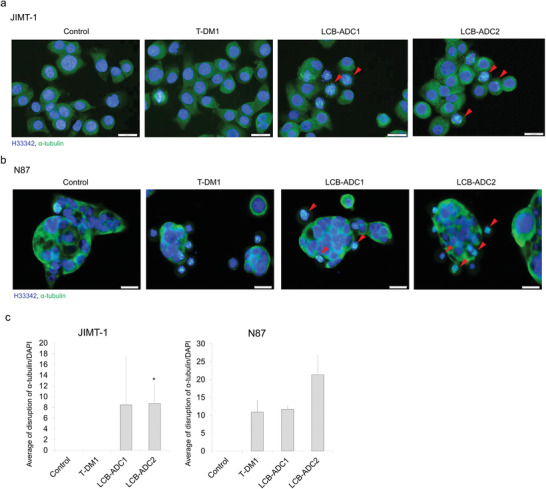
In vitro microtubule interruption by LCB‐ADCs. a) JIMT‐1 and b) N87 cells were treated with T‐DM1, LCB‐ADC1, or LCB‐ADC2 at 0.06 µg mL^−1^ for 16 or 24 h and stained with anti‐*α*‐tubulin (blue, Hoechst‐stained nuclei; green, Alexa 488‐stained *α*‐tubulin, scale bar, 10 µm). c) Ratio of tubulin disrupted cell per H33342 stained cells (*n* = 3). Data values are a mean ± standard deviation. *p < 0.05 (T‐DM1 vs LCB‐ADC2).

Among the analogs of dolastatin, MMAF exerts known antineoplastic and antitubulin effects by inhibiting cell division via the disruption of tubulin polymerization. MMAF was used as the toxic component of our LCB‐ADCs as it was designed to be much more active when delivered into cells with an antibody. It harbors a binding site in its C‐terminal carboxyl group for a dipeptide linker attachment to enzymes capable of catalyzing drug release, and it can modulate the efficacy, potency, and tolerability of the drug. To evaluate the effect of our novel LCB‐ADCs on the cell cycle distribution caused by the payload tubulin inhibitors, human breast cancer JIMT‐1 cells and human gastric cancer N87 cells were treated and then analyzed by flow cytometry (**Figure** **3**). Trastuzumab emtansine (T‐DM1 or Kadcyla, Genentech), that pairs trastuzumab (Herceptin, Genentech) with the microtubule inhibitor DM1 containing a nonreducible thioether linker,^[^ ^35^ ^]^ and was approved in 2013 as a second‐line treatment for HER2‐positive metastatic breast cancer, was used as the reference ADC for comparison.^[^ ^19^ ^]^ HER2 expression in the JIMT‐1 and N87 cells was first evaluated by immunocytochemistry (ICC, Figure 3a). LCB‐ADC1, 2, and 3 induced G2/M phase arrest to 47.7%, 63.8%, and 68.5% of the cell populations after 24 h in the HER2 low expressing JIMT‐1, cells, respectively, and they maintained this arrest for up to 72 h unlike the control or T‐DM1 treated cells (Figure 3b). In contrast, the N87 high expressing HER2 cells showed a similar induction of G2/M phase arrest by all three LCB‐ADC drugs and by T‐DM1 (Figure 3c). T‐DM1 did not induce G2/M phase arrest in the JIMT‐1 cells until 72 h post‐treatment, and this was identical to the control. This is consistent with previous evidence that T‐DM1 is effective only in cells expressing high levels of HER2.^[^ ^26^ ^]^ Moreover, while T‐DM1 showed different effects in the two cell lines we tested, our LCB‐ADCs showed similarly potent effects in all of the cells regardless of their HER2 expression level (Figure 3b). Furthermore, LCB‐ADC2 and LCB‐ADC3, both having a DAR of 4, showed a greater effect on G2/M phase arrest than LCB‐ADC1 in JIMT‐1 cells. This suggested that a higher HER2 expression level allows for a higher accessibility of the HER2‐ADCs, leading to a more readily disrupted cell cycle regardless of the DAR homogeneity. In cells where access to HER2‐ADCs is low due to low HER2 expression, the homogeneity of the ADC and the effective delivery of the payload drug will be crucial. The LCB‐ADCs disrupted tubulin polymerization in the JIMT‐1 cell line within 24 h, whereas all of the drugs in the experiment including T‐DM1 could exert this effect in the N87 cells. Abnormal tubulin polymerization induced by the payload drugs of the ADCs was visualized by tubulin staining (**Figure** **4**, red arrow), and the resulting mitotic arrest and eventual death of the cells was captured using live cell imaging (Video S1, Supporting Information).

### 2.3 LCB‐ADCs Effectively Target HER2‐Positive Xenograft Tumors That Are Resistant to T‐DM1

**Figure 5:**
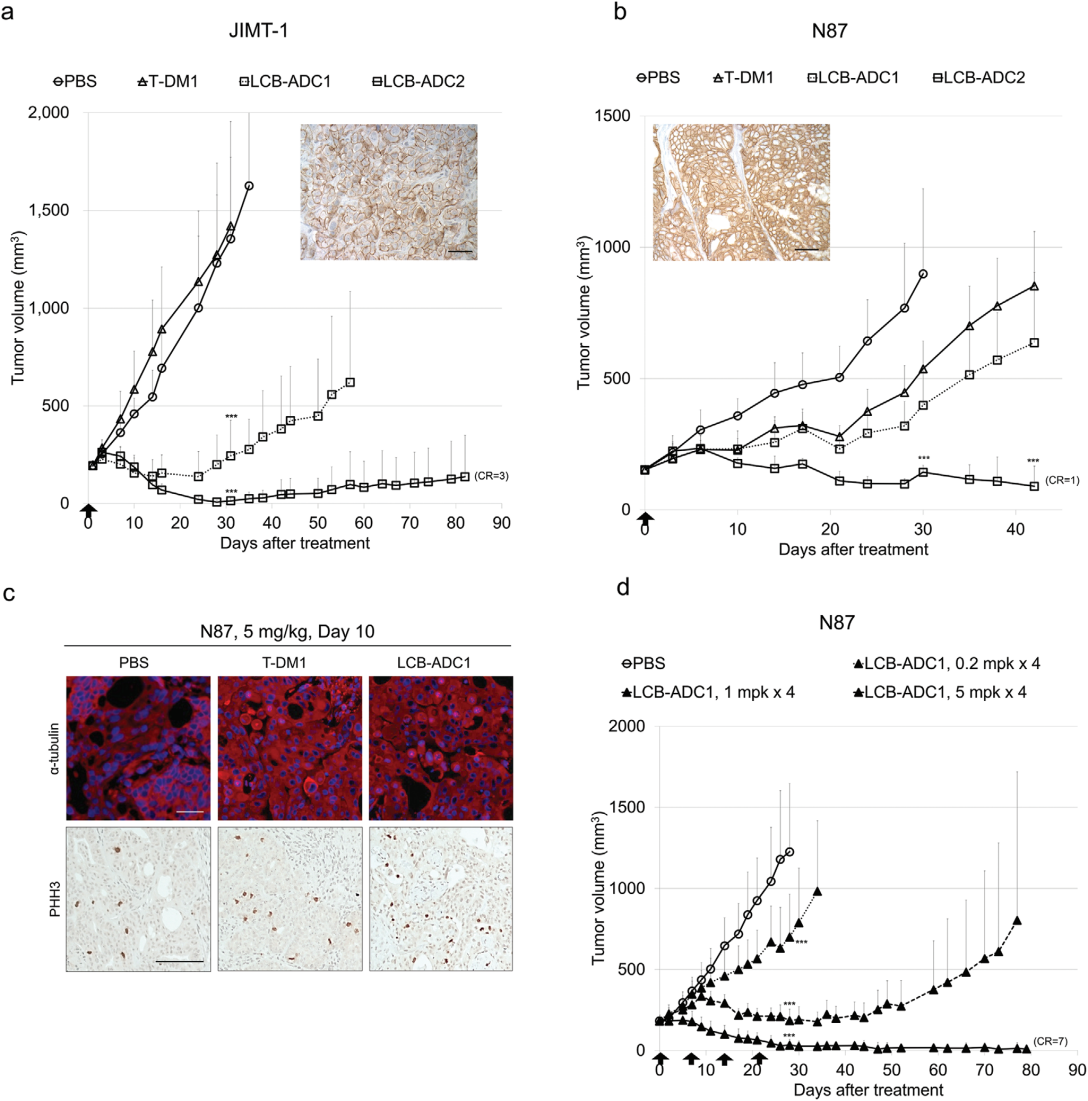
Therapeutic efficacy of LCB‐ADCs in HER2‐positive CDX models and microtubule interruption and mitotic arrest in CDX tumor tissue. a) Effect of LCB‐ADC1, LCB‐ADC2, T‐DM1 in JIMT‐1 cells. Drugs were i.v. injected at 2 mg kg^−1^ once (↑), *n* = 5. Data values are the mean ± standard deviation. ***p < 0.001 (T‐DM1 vs LCB‐ADC1 or 2). b) Effect of LCB‐ADC1, LCB‐ADC2, T‐DM1 in N87 cells. Drugs were i.v. injected at 2 mg kg^−1^ once (↑), *n* = 5. Data values are the mean ± standard deviation. ***p < 0.001 (T‐DM1 vs LCB‐ADC 2). HER2 expression in tumor tissue was detected by IHC (brown, DAB‐stained HER2; blue, hematoxylin‐stained nuclei, scale bar, 10 µm). c) In vivo microtubule interruption and mitotic arrest by LCB‐ADC1. Mice bearing N87 tumors were i.v. administered with T‐DM1, LCB‐ADC1 at 5 mg kg^−1^ once. After 10 d, the tumors were isolated, fixed, and stained with anti‐*α*‐tubulin (upper, blue, Hoechst‐stained nuclei; red, Alexa 594‐stained *α*‐tubulin, scale bar; 10 µm) or antiphosphohistone H3 (PHH3) (lower, blue, hematoxylin‐stained nuclei; brown, 3,3′‐diaminobenzidine (DAB)‐stained PHH3, scale bar, 100 µm). d) Effect of LCB‐ADC1 in N87 cells. Drugs were i.v. injected at 0.2, 1 or 5 mg kg^−1^, once a week for 4 weeks (↑), *n* = 8. Data values are the mean ± standard deviation. *** ***p*** < 0.001 (T‐DM1 vs LCB‐ADC1).

We conducted in vivo experiments and found that T‐DM1 displayed a moderate effect in an N87 xenograft tumor‐bearing mouse model, but not in the counterpart JIMT‐1 model, likely due to the different HER2 expression levels (**Figure** **5**a,b). In contrast to T‐DM1, the LCB‐ADCs showed potent in vivo efficacy in both the JIMT‐1 and N87 xenograft models as well as a DAR‐dependency between LCB‐ADC1 and LCB‐ADC2. In the JIMT‐1 model, tumor growth was 96% inhibited by LCB‐ADC1, 115% by LCB‐ADC2, and tumor‐free complete remission (CR) was observed in three out of five mice in the LCB‐ADC2‐treated group at the end point of the experiment (Figure 5a). In the N87 model, tumor growth was inhibited by 48%, 67%, and 101% by T‐DM1, LCB‐ADC1, LCB‐ADC2, respectively, and one out of five mice were tumor‐free in the LCB‐ADC2‐treated group at the end point (Figure 5b). Moreover, repeated treatments with LCB‐ADC1 produced very effective tumor growth inhibition, with tumor‐free CR observed in seven out of eight animals at the end point (Figure 5d).

T‐DM1 showed intermediate efficacy in the high HER2‐expressing N87 tumor model but had no effect in the low expressing JIMT‐1 model, which had comparable tumor growth to the control group (Figure 5). These results indicated that while LCB‐ADCs have very potent antitumor efficacy against low expressing HER2 lesions that are resistant to T‐DM1, they are also more effective than T‐DM1 against high expressing HER2 tumors.

**Figure 6:**
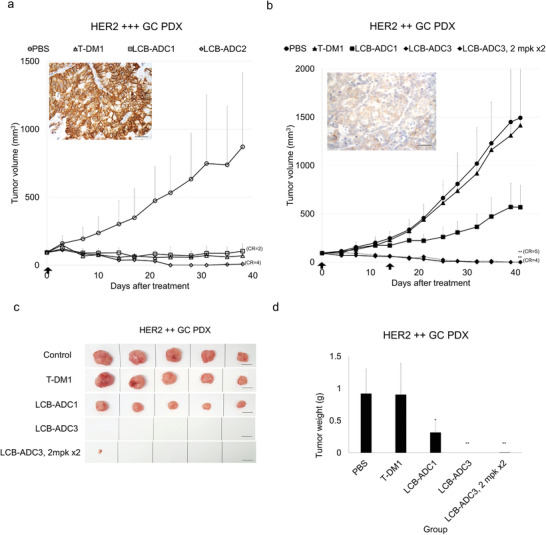
Therapeutic efficacy of LCB‐ADCs in HER2‐positive GC PDX models. a) Effect of LCB‐ADC1, LCB‐ADC2, T‐DM1 in an HER2 +++ GC PDX model. The drugs were i.v. injected at 5 mg kg^−1^ once (↑), *n* = 5. b) Effect of LCB‐ADC1, LCB‐ADC3, T‐DM1 in an HER2 ++ GC PDX model. Drugs were i.v. injected at 5 mg kg^−1^ twice (↑), n = 5. Data values are a mean ± standard deviation. **p < 0.01 (T‐DM1 vs LCB‐ADC3, single or repeated treatment). HER2 expression in tumor tissues was detected by IHC (brown, DAB‐stained HER2; blue, hematoxylin‐stained nuclei, scale bar, 10 µm). c,d) Mice bearing HER2 ++ GC patient‐derived tumors were i.v. administered with LCB‐ADC1, LCB‐ADC3, or T‐DM1 at 5 mg kg^−1^, every other week for 4 weeks, *n* = 5. At the end of the experiment, c) tumors were isolated, photographed (scale bar, 1 cm) and d) weighed. Data values are mean ± standard deviation. *p < 0.05 (T‐DM1 vs LCB‐ADC1), **p < 0.01 (T‐DM1 vs LCB‐ADC3).

**Figure 7:**
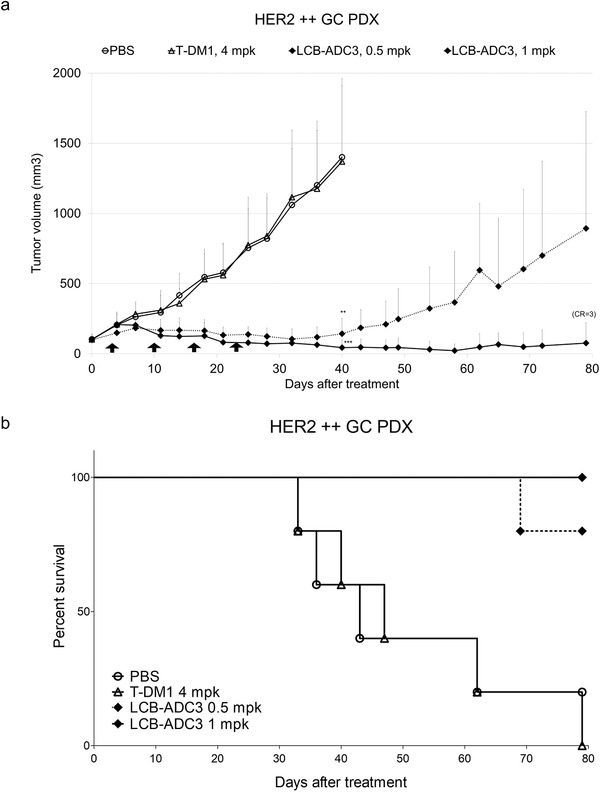
In vivo efficacy of LCB‐ADC3 at a low dose in an HER2 ++ GC PDX model. a) Tumor growth delay curves and b) survival rate when the tumor volume reached 1500 mm^3^. Mice bearing patient‐derived tumors were i.v. administered LCB‐ADC3 (0.5 or 1 mg kg^−1^) or T‐DM1 (4 mg kg^−1^), weekly for 4 weeks (↑), *n* = 5. Data values are the mean ± standard deviation. **p < 0.01 (T‐DM1 vs LCB‐ADC3, 0.05 mg kg^−1^), ***p < 0.001 (T‐DM1 vs LCB‐ADC3, 1 mg kg^−1^).

To determine whether our linker‐drug technology may be clinically applicable and thus potentially address one of the most critical limitations of existing HER2‐ADCs, the in vivo efficacy of our LCB‐ADCs was evaluated in patient‐derived xenograft (PDX) tumor models, which were considered to be the most relevant model for patients.^[^ ^36^ ^]^ PDX models prepared with human GC tissues in which HER2 expression was relatively high (HER2 +++ GC PDX) or low (HER2 ++ GC PDX)^[^ ^37^ ^]^ were treated with T‐DM1 or LCB‐ADCs (**Figure** **6**a,b). In the HER2 +++ model, tumor growth was inhibited by 103%, 99%, and 111% by T‐DM1, LCB‐ADC1, and LCB‐ADC2, respectively, and two out of five mice were tumor‐free in the LCB‐ADC2‐treated group at day 38 (Figure 6a). In the HER2 ++ GC PDX model, tumor growth was inhibited by 66% using repeated LCB‐ADC1 treatments at 5 mg mL^−1^, 107% by repeated treatments with LCB‐ADC3 at 5 mg mL^−1^, and tumor‐free CR was observed in all mice in the LCB‐ADC3‐treated group at the end point (Figure 6b). In addition, repeated doses of LCB‐ADC3 at 2 mg mL^−1^ produced sufficient tumor growth inhibition (107%), with tumor‐free CR observed in four out of eight animals at the end point (Figure 6b). In the HER2 +++ GC PDX model, T‐DM1 showed a potent therapeutic efficacy as expected. However, the LCB‐ADCs displayed an equally potent efficacy to T‐DM1 in this model with one treatment (Figure 6a), but it showed a more prolonged efficacy than T‐DM1 over a repeated course (Figure S1a, Supporting Information). Although T‐DM1 has been successfully translated to the clinic for HER2‐positive cancer patients, its lack of efficacy in tumor cells expressing relatively low HER2 levels is an important unmet clinical need.^[^ ^26^ ^]^ T‐DM1 did not have any effect in the HER2 ++ GC PDX model but the LCB‐ADCs still showed dramatic therapeutic efficacy in these mice. LCB‐ADC1 inhibited the growth of the tumors by 66% and LCB‐ADC3, after pegylation of LCB‐ADC2 in order to test a clinically suitable candidate, produced completely tumor‐free mice in all cases, which was confirmed by measurement of the tumor weights in each group and by macrography (Figure 6). LCB‐ADC3 was further examined to see if it was still effective at a lower dosage or as a single treatment in the HER2 ++ GC PDX model. A lower dose of LCB‐ADC3 displayed a strong therapeutic efficacy whereby up to three out of five mice became tumor‐free following repeated treatments at a lower concentration of this drug and there was a greatly increased survival rate (**Figure** **7**). Moreover, LCB‐ADC3 showed sufficient antitumor potency with a tumor inhibition rate of 106% and CR in all HER2 ++ GC PDX mice after only a single treatment (Figure S1b, Supporting Information) These results clearly demonstrated that the HER2‐targeting LCB‐ADCs produced by our linker‐drug technology are clinically applicable and address some of the significant shortfalls with current HER2‐targeting therapeutics.

### 2.4 LCB‐ADCs Evokes Mitotic Arrest in HER2‐Overexpressing and ‐Low Expressing Cell‐Derived Xenograft (CDX) and PDX Model*s*

**Figure 8:**
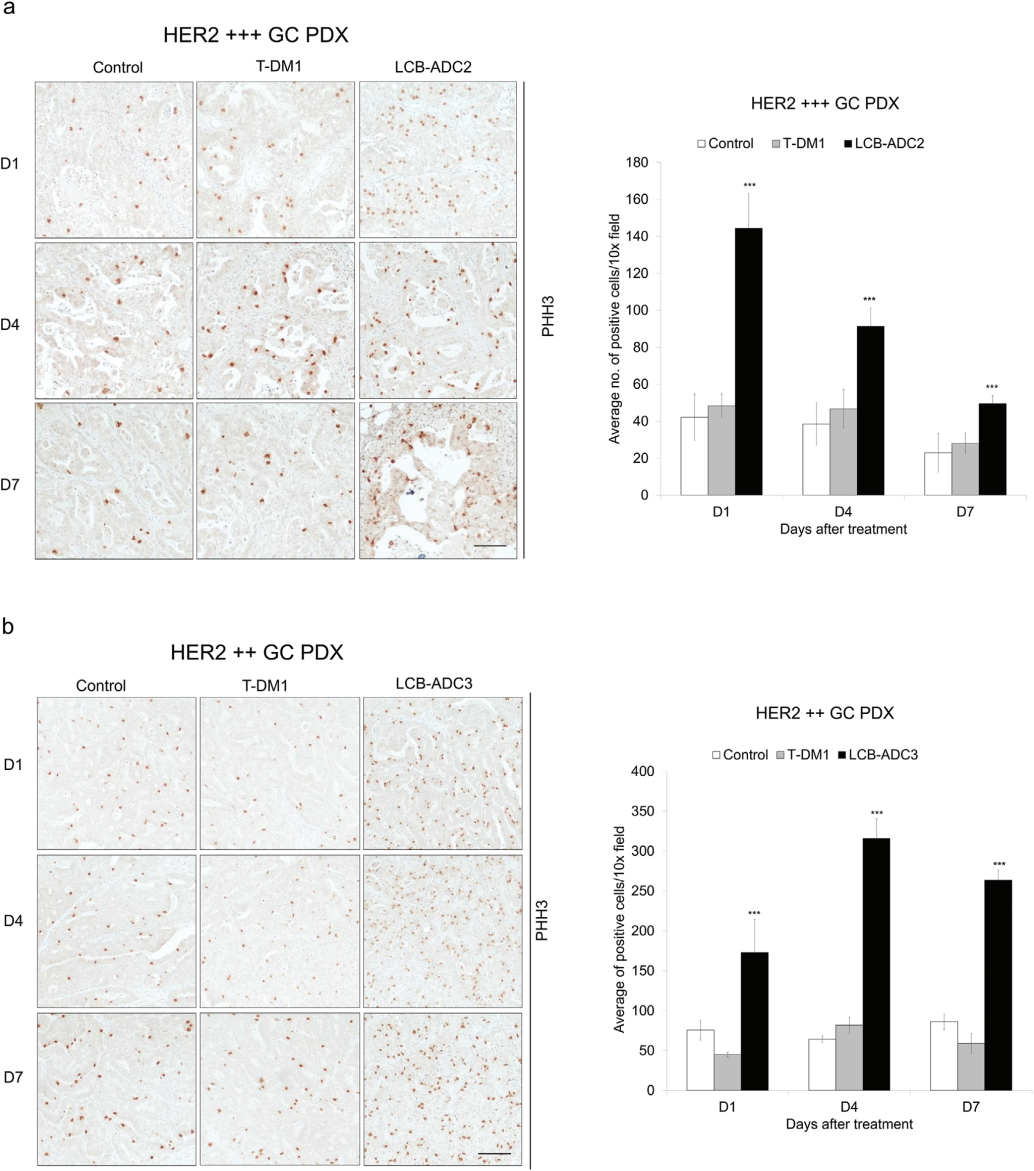
Mitotic arrest of HER2‐positive GC PDX tumor tissue. a,b) Mice bearing HER2‐positive PDX tumors were i.v. administered with a) LCB‐ADC2, b) LCB‐ADC3 or T‐DM1 at 5 mg kg^−1^ once, *n* = 5. After 1, 4, and 7 d, the tumors were isolated, fixed, and stained with antiphosphohistone H3 (PHH3) (blue, hematoxylin‐stained nuclei; brown, DAB‐stained PHH3, scale bar, 100 µm). Data values are a mean ± standard deviation. ***p < 0.001 (T‐DM1 vs LCB‐ADC2 or 3).

Cells under mitotic arrest with abnormal tubulin polymerization and the presentation of phosphohistone H3 (PHH3) induced by the intracellular influx of the payload drug were observed in the N87 tumor tissues of HER2‐ADC treated mice, and this effect was more significantly augmented by LCB‐ADC1 than by T‐DM1 (Figure 5c). When N87 xenograft mice were periodically treated with LCB‐ADC1 with a DAR of 2, sufficient inhibition was achieved at a lower dose of 1 mg kg^−1^, and dose‐dependency was verified (Figure 5D). Cells arrested in mitosis (expressing PHH3) due to HER2‐ADC exposure were observed in these PDX models (**Figure** **8**). Considerable changes of body weight were not observed in this experiment, indicating that no mice suffered from severe toxicity (Figure S2, Supporting Information). These results further indicated that LCB‐ADCs displayed an efficient anticancer effect in HER2‐expressing tumors even at a low level of HER2, and strongly suggest that the intended advantages of LCB‐ADCs, such as increased linker stability in the bloodstream, an accurate DAR and reliable homogeneity, and an improved PK profile can be observed in xenograft tumor models.

## 3 Discussion and Conclusion

ADCs have been continuously investigated as a significant breakthrough technology for targeted therapeutics against various cancers, with nine such drugs having current approval for clinical use and more than 40 kinds of ADCs currently in clinical trials.^[^ ^38^ ^]^ The most interesting ADC to emerge has been T‐DM1 (Kadcyla) that consists of an HER2 antibody (trastuzumab) conjugated with DM1 that has been approved by the US FDA as a treatment for breast cancer.^[^ ^17^ ^]^ Despite the development of T‐DM1 for cancers that are resistant to trastuzumab, many patients treated with this drug ultimately progress to late stage disease, and some HER2‐positive patients have no or a low response.^[^ ^39^ ^]^ In the previous TDM4450g trial, 46% of metastatic breast cancers did not respond to T‐DM1.^[^ ^40^ ^]^ Moreover, T‐DM1 was found not to improve the objective response in the EMILIA trial in a second‐line setting among patients treated with trastuzumab and taxane, i.e., in 228 (66%) of the 397 subjects.^[^ ^19^ ^]^ Furthermore, the recent GASTBY study has reported that T‐DM1 was not superior to taxane in HER2‐positive advanced GC patients receiving previous HER2‐targeting therapy, in which the median overall survival was 7.9 months with T‐DM1 and 8.6 months with taxane.^[^ ^26^ ^]^ Although Enhertu was recently approved by the FDA for treating HER2‐positive breast cancers, about 40% of metastatic breast cancer cases showed a low or zero response to this ADC in the phase II DESTINY‐Breast01 trial.^[^ ^23^ ^]^ In addition, although Enhertu is comprised of a maleimide tetrapeptide‐based cleavable linker and has a higher DAR of ≈8,^[^ ^41^ ^]^ it is a less elaborate molecule but similar to T‐DM1. In contrast, our LCB‐ADCs harbor a cleavable linker and have a precise DAR. An expected feature of LCB‐ADCs is fewer adverse effects because LCB‐ADCs did not show severe weight change and this technology thus represents a promising new ADC‐platform for a variety of targets.

Retrospective analyses of two phase II trials of T‐DM1 have indicated that lower response rates were achieved in patients with lower HER2 levels,^[^ ^41^, ^42^ ^]^ suggesting that the actual HER2 expression level is critical for this drug to work, even in the subset of cancers that are judged to be HER2‐positive. The major limitations of T‐DM1 are thought to be the inexact DAR control and its noncleavable linker.^[^ ^17^ ^]^ This prompted us to search for methods that would enhance the linker stability and produce harmonious fabrication (Figure 1). We expected that breast and gastric cancers that would not respond to T‐DM1 could be targeted successfully with better next‐generation anti‐HER2 ADCs and found this to be the case with our LCB‐ADCs. These novel agents displayed overwhelming efficacy in both in vitro and in vivo systems, including in the T‐DM1‐none‐responsive breast cancer line JIMT‐1, GC line N87, and in gastric PDX models with low HER2 expression (HER2++) (Figures 3, 5, 6, and 8). Substantial G2/M arrest and rapid cell death was caused in all cases by the effective actions of MMAF, as observed via live imaging (Video S1, Supporting Information) and histologically increased PHH3 levels (Figures 5 and 7). Of note in particular, complete remission was observed in our xenograft models following LCB‐ADC treatments, i.e., the N87, JIMT‐1, and HER2++ PDX models that were not responsive to the established ADC, T‐DM1.

In contrast with T‐DM1, LCB‐ADCs contain a cleavable linker that is designed to facilitate a higher efficacy through various mechanisms, including the bystander effect,^[^ ^42^ ^]^ and they were constructed to achieve a consistent DAR of 2 or 4, that is, stable in plasma and thus significantly lengthening the half‐life of the drug (Figure 2). We observed that our LCB‐ADCs had a significantly improved antitumor efficacy in both HER2 overexpressing and low‐expressing cases. This greater anticancer effect of LCB‐ADCs may also be augmented by the bystander effects of MMAF, which is membrane‐permeable and can therefore enter and kill neighboring cells unlike other agents used in ADCs, such as monomethyl auristatin E (MMAE),which remains trapped in the cell.^[^ ^27^ ^]^ As another example, lysine‐MCC (4‐(N‐maleimidomethyl))‐DM1,which is formed by the proteolytic degradation of T‐DM1 in the lysosomes, also cannot cross the cell membrane and T‐DM1, and therefore cannot induce a bystander effect.^[^ ^21^ ^]^ Both DM1 and the auristatins are highly cytotoxic payloads and cause cell death by inhibiting tubulin polymerization.^[^ ^42^ ^]^ Antibody‐dependent cellular cytotoxicity (ADCC) likely contributes substantially to the efficacy of trastuzumab and T‐DM1 in vivo.^[^ ^32^, ^43^ ^]^ The a novel anti‐HER2 antibody, HT‐19 component of a novel anti‐HER2 ADC, XMT‐1522 may also evoke ADCC.^[^ ^44^ ^]^

The intensity of HER2 overexpression in GCs is typically scored using immunohistochemistry (IHC) staining (3+ or 2+) and fluorescence in situ hybridization (FISH).^[^ ^43^ ^]^ Most HER2‐targeting therapies are given to histologically HER2‐positive patients (IHC3+ or IHC2+/FISH‐positive cases), but HER2 scoring as a diagnostic process in these patients is still controversial.^[^ ^44^, ^45^ ^]^ The HER2 status guidelines were announced following the results of the Trastuzumab for Gastric Cancer (ToGA trial), which showed that this drug significantly increased the survival time of patients when administered in combination with 5‐fluorouracil or capecitabine and cisplatin, and this status has since become an important predictor of treatment outcomes for breast as well as stomach cancer.^[^ ^43^, ^46^ ^]^ Based on these findings, a combination of trastuzumab with other chemotherapeutics in patients with HER2 positive GC has become the primary standard therapy. Despite the availability of various HER2‐targeting agents such as trastuzumab, pertuzumab, lapatinib, and T‐DM1, which have been approved for treating patients with HER2‐positive tumors worldwide, the trastuzumab monoclonal antibody against the HER2 protein is the only targeted treatment used for patients with HER2‐positive GC.^[^ ^43^ ^]^

In conclusion, we here describe a novel and highly effective linker‐drug technology that can be adopted as a platform technology for a broad range of ADCs and has the potential to greatly enhance their efficacy against a wide range of cancer targets. Our LCB‐ADCs targeting HER2 show a stronger inhibitory effect against breast and GC cells than T‐DM1, including those that are sensitive to T‐DM1. In addition, these novel agents show significant efficacy against GC cells with primary or acquired resistance to T‐DM1 in vitro, and against T‐DM1‐resistant breast cancer and GC xenografts in vivo. A full clinical evaluation of LCB‐ADCs in patients with HER2‐positive breast or gastric cancer is warranted.

## 4 Experimental Section

### Construction, Expression, and Purification of the Herceptin‐CaaX Body

ModifiedHerceptin antibodies were generated using standard polymerase chain reaction (PCR) cloning protocols. Generally, Herceptin‐CaaX body plasmids were constructed by inserting a DNA sequence encoding a CaaX motif (G_7_CVIM) into the C‐terminus of the light chain encoded in the pNATABH::Herceptin light chain (LC) plasmid. HEK293 E cells were cultured in dulbecco modified eagle medium (DMEM)/10% fetal bovine serum (FBS) media on 150 mm plates (#430599; Corning, Glendale, AZ) until reaching 70–80% confluency. Then, 13 mg of DNA and 26 mg of polyethylenimine (PEI) (#23966, Polysciences, Warrington, PA) were mixed at a ratio of 1:2, incubated at room temperature for about 20 min, and then added to the HEK293E cells. After 16–20 hour, the media was replaced with serum free media (DMEM (#SH30243.01; Hyclone Thermo, Marlborough, MA) without FBS and the supernatant was collected every 2 or 3 day. The supernatants were filtered through a 0.22 µm top‐filter (#PR02890, Millipore, Milford, MA) and then bound to 500 mL of protein A beads (#17‐1279‐03; GE healthcare, Danderyd, Sweden) packed in a 5 mL column. Using a peristaltic pump, overnight binding was performed at 0.9 mL min^−1^ at 4 °C. The column was washed with at least 100 mL of phosphate‐buffered saline (PBS, #70011, Gibco, Waltham, MA). Bound protein was then eluted with 0.1 m glycine‐HCl (#G7126; Sigma, St. Louis, MO) into six fractions and neutralized with 1 m Tris (#T‐1503; Sigma, St. Louis, MO) (pH 9.0). The protein was quantified and two or three fractions containing the protein were collected and concentrated with Amicon Ultra filter units (#UFC805024; Millipore). The buffer was changed about ten times with PBS (#70011; Gibco). The protein product was confirmed to be Herceptin‐HC‐GCVIM amino acid sequence or Herceptin‐LC‐GCVIM amino acid sequence by western blot. To identify a protein band containing Herceptin, ImmunoPure peroxidase conjugated goat antihuman IgG Fc (#31413; Pierce, Waltham, MA) was used. Upon purification, Herceptin‐CaaX was obtained from 1 L of cell culture medium. The Herceptin‐CaaX body products were also analyzed with an Agilent bioanalyzer. Briefly, 8 mL of purified protein sample (≈1 mg mL^−1^) was analyzed using the Agilent Protein 230 Kit (5067‐1515; Agilent Technologies, Santa Clara, CA). The protein sample was then separated into two fractions to which 2 mL of nonreducing buffer or reducing buffer was then added. The sample was next heated at 95–100 °C for 5 min and cooled with ice to 4 °C. After a spin‐down, 84 mL of deionized water was added to the sample and vortexed. Thereafter, the sample was loaded and analyzed with the kit per the manufacturer's instructions.

### Prenylation

The prenylation reaction mixture was prepared with the antibodies and reacted at 30℃ for 12 h. The reaction mixture was comprised of a buffer solution (50 × 10^−3^ m Tris‐HCl (pH 7.4), 5 × 10^−3^ m MgCl_2_, 10 × 10^−6^ m ZnCl_2_, 5 × 10^−3^ m dithiothreitol (DTT)) containing 24 × 10^−6^ m antibody, 200 × 10^−9^ m FTase (#344145, Calbiochem, Milford, MA), and 1 × 10^−3^ m LCB14–0606 (LegoChemBiosciences, Inc., US2012/0308584). After the reaction was completed, the prenylated antibody was purified with a G25 Sepharose column on an AKTApurifier (AF fast protein liquid chromatography systems) (GE healthcare), which was equilibrated with PBS buffer solution. The prenylated antibody (final concentration: 12 × 10^−6^ m) was then treated with 1 × 10^−3^ m CuSO_4_ and reacted at 30℃ for 3 h in order to be reoxidized. After this reaction was completed, 2 × 10^−3^ m (final concentration) ethylenediaminetetraacetic acid (EDTA) was added and the mixture was kept at 30 °C for 30 min while being gently stirred. The resultant preparation was then purified by fast protein liquid chromatography (FPLC).

### Drug Conjugation

An oxime bond formation reaction mixture between the prenylated antibody and linker‐toxin was prepared by mixing 100 × 10^−3^ m Na‐acetate buffer (pH 4.5, 10% DMSO; dimethyl sulfoxide), 12 × 10^−6^ m antibody, and 360 × 10^−6^ m linker‐toxin and gently stirred at 30 ℃. After the reaction had proceeded for 24 h, FPLC (AKTA purifier, GE healthcare) was conducted to remove any excess amounts of small molecules used in the reaction and the protein fractions were collected to be subsequently concentrated. Depending on the type of linker‐payload, the DARs of LCB‐ADC1, LCB‐ADC2, and LCB‐ADC3 were 2, 4, and 4, respectively.

### In Vivo Rat Pharmacokinetics of Herceptin and LCB‐ADC1

To confirm the pharmacokinetics of Herceptin and LCB‐ADC1 at the time of intravenous administration of a single dose to a rat, the following experiment was performed. Herceptin or LCB‐ADC1 were intravenously administered to female rats (dose: 3.0 mg kg^−1^) and 0.4 mL of blood was collected from the jugular vein using a 1 mL syringe (25 gauge) treated with Heparin (85 IU mL^−1^, 35 µL) at predetermined times (3 min; 1, 3, and 6 h; and 1, 2, 3, 4, 7, 9, 14, 17, 21, and 28 d after administration). The blood samples were placed a micro tube, treated with a roll mixer for several minutes, and then centrifuged at 14 000 rpm for 5 min, thereby separating the plasma. The separated plasma was put into a micro tube and stored in a deep freezer until analysis. The test materials in the plasma were measured using liquid chromatography–mass spectrometry (LC‐MS). PK analysis was conducted using compartment model 2 using Phoenix WinNonlin (ver. 6.3, Pharsight) and the drug‐antibody ratio (DAR) was quantified by LC‐MS/MS after separating the LCB‐ADC1 using protein A beads.

### In Vivo Monkey Pharmacokinetics of Herceptin and LCB‐ADC1

To determine the pharmacokinetics of Herceptin and LCB‐ADC1 in the monkey, Herceptin and LCB‐ADC1 were intravenously administered into female monkeys, respectively (dose: 3.0 mg kg^−1^). About 1.5 mL of blood was collected from the cephalic or femoral vein at predetermined times (30 min; 3, 7, 12, and 24 h (2 d); 3, 4, 5, 6, 11, 15, 22, 29, and 36 d after administration), put into a tube filled with anticoagulant (EDTA‐K2), stored in a wet‐ice/Kryorack and then centrifuged at 3000 rpm for 10 min in a cold‐storage state, thereby separating the plasma. The separated plasma was dispensed into a microtube and stored in a deep freezer until analysis. The test materials in the plasma were measured using LC‐MS. PK analysis was performed with compartment model 2 using Phoenix WinNonlin (ver. 6.3, Pharsight), and in order to quantify the drug‐antibody ratio, free MMAF was quantified by LC‐MS/MS after separating LCB‐ADC1 using protein A beads and treatment with *β*‐glucuronidase.

### Cell Culture

Human gastric carcinoma N87 cells (American Type Culture Collection, ATCC no.CRL‐5822, Manassas, VA) were maintained in Roswell Park Memorial Institute (RPMI)1640 medium (Gibco‐Invitrogen, Carlsbad, CA). Human breast carcinoma JIMT‐1 cells (Deutsche Sammlung von Mikroorganismen und Zellkulturen GmbH, DSMZno. ACC 589, Inhoffenstraße, Braunschweig) were maintained in DMEM medium (Gibco‐Invitrogen). Both media were supplemented with 10% fetal bovine serum (Gibco‐Invitrogen) and 1% penicillin/streptomycin (Gibco‐Invitrogen) and the cells were grown under a humidified atmosphere of 5% CO_2_ at 37 °C. The cells were tested for possible mycoplasma contamination using MycoAlert PLUS Mycoplasma Detection Kit (Lonza Walkersville, Inc., Walkersville, MD).

### Cell Cycle Distribution

JIMT‐1 and N87 cells were plated in a 60 mm tissue culture dish at a density of 5 × 10^5^ cells plate^−1^ and incubated overnight. Cells were treated with T‐DM1 (Kadcyla, 10172048, Roche, Basel, Switzerland) or LCB‐ADCs (provided by LegoChem Bioscience) at a dose of 0.25 µg mL^−1^ based on the amount of antibody for 24–72 h. The cells were collected and suspended in 100% cold‐ethanol for fixation, stained with propidium iodide (PI), and then the cell cycle distribution was analyzed by flow cytometry (BD FACSCancto II).

### Immunocytochemistry

N87 and JIMT‐1 cells were cultured in the appropriate medium supplemented with 10% fetal bovine serum and 1% penicillin/streptomycin at 37 °C under 5% CO_2_. Upon confluency, the cells were detached from the flask by trypsin–EDTA, centrifuged and resuspended in media. Approximately 5 × 10^4^ cells mL^−1^ were then transferred onto 12 mm round coverslips in a 24‐well plate and incubated for 24 h. For assessment of microtubule interruption, they were treated with T‐DM1, LCB‐ADC1, or LCB‐ADC2 at 0.25 µg mL^−1^ and incubated for 24 h. Then, they were fixed with 4% formaldehyde and treated with 0.5% Triton X‐100. After thorough washing with PBS, the cells were incubated with anti‐HER2/ErbB2 rabbit polyclonal primary antibody (#2242S; Cell Signaling, Danvers, MA), anti‐*α*‐tubulin rabbit monoclonal primary antibody (#2125S; Cell Signaling), and Alexa 594 or 488 antirabbit IgG (Jackson Immuno Research, West Grove, PA). The cells were incubated with Hoechst33342 (H33342; Sigma‐Aldrich, St. Louis, MO) for 10 min at room temperature. After washing with PBS, the coverslips were mounted on slides (Vector Laboratories, Inc., Burlingame, CA). Fluorescence imaging was performed under a DP71 microscope (Olympus, Tokyo, Japan) equipped with two band‐pass filters with a center wavelength.

### In Vivo Tumor Growth Delay

CDX and PDX tumor models using male athymic nude mice (BALB/c‐nude (6 weeks old); Japan Shizuoka Laboratory Center (SLC),Hamamatsu, Japan) were used for the examination of in vivo therapeutic efficacy. A suspension of 3 × 10^6^ cells for the CDX model or a 3 × 3 × 3 mm^3^ tissue block for the PDX model were implanted subcutaneously into the right hind leg of the mouse. The mice bearing xenograft tumors grown to 80 to 120 mm^3^ were pair matched according to the tumor volume into the experimental and control groups (*n* = 5 per group).^[^ ^37^ ^]^ T‐DM1 (Kadcyla, 10172048, Roche, Basel, Switzerland), LCB‐ADC1, LCB‐ADC2, and LCB‐ADC3 were intravenously (i.v.) administrated through the tail vein, and the dose was a single 2 or 5 mg kg^−1^ or repeated 5 mg kg^−1^, once a week for 4 weeks or every other week for 4 weeks. The doses of all drugs were adjusted to the equivalent amount of the antibody. The tumor volumes and body weights were monitored during the entire experiment. The tumor volumes were calculated using the formula volume = (length × width^2^) × 0.5. The results are expressed as a mean ± standard deviation. An objective response was defined using the Response Evaluation Criteria In Solid Tumors system as a complete response (CR; disappearance of all target lesions), PR (at least a 30% decrease in the longest diameter of the target lesions), stable disease (SD, neither sufficient shrinkage to qualify as a PR nor sufficient increase to qualify as progressive disease, PD), and PD (at least a 20% increase in the size of the target lesion).

### IHC

For immunohistochemical staining of HER2, tumor tissues from the N87 CDX, JIMT‐1 CDX, or PDX models were fixed with 4% paraformaldehyde, embedded in paraffin and sectioned. Immunohistochemical staining was conducted with the anti‐HER2/neu (4B5) rabbit monoclonal primary antibody (#790‐4493; Roche, Basel, Switzerland), and the stained sections were analyzed under a DP71 microscope (Olympus, Tokyo, Japan). The tumor tissues were counterstained with hematoxylin.

### Statistical Analysis

Sample sizes were not predetermined. All data are presented as a mean ± SD (standard deviation) from at least three separate experiments. Means were compared using a two‐tailed *t*‐test. *P* values were obtained by Microsoft Excel 365. *P* values are represented as *P < 0.05, **P < 0.01 or ***P < 0.001, which are considered to indicate significant differences.

### Study Approval

All animal experiments were performed following a protocol approved by the Institutional Animal Care and Use Committee of the Asan Institute for Life Science (2014‐12‐168, 2015‐12‐126). All of the patients provided signed informed consent. This study was approved by the Institutional Review Board of Asan medical center (2010–0618).

## Conflict of Interest

Y.‐H.P. has served as a new drug research team leader for LegoChem Biosciences, Inc., The other authors declare no conflicts of interest in relation to this study.

## Supporting information

Supporting Information

Supplemental Video 1

Supplemental Video 2

Supplemental Video 3

Supplemental Video 4

## Data Availability

The data that support the findings of this study are available from the corresponding author upon reasonable request.
