# Supplementary material for: An Elaborate New Linker System Significantly Enhances the Efficacy of an HER2‐Antibody‐Drug Conjugate against Refractory HER2‐Positive Cancers
Source: Adv Sci (Weinh). 2021 Oct 18;8(23):2102414. doi: 10.1002/advs.202102414 (PMC8655175; doi:10.1002/advs.202102414)
Supplement: Supplementary file 1 — Supporting Information [file ADVS-8-2102414-s005.pdf]

© 2021 Wiley-VCH GmbH

ADVANCED SCIENCE
Open Access

Supporting Information

for *Adv. Sci.,* DOI: 10.1002/advs.202102414

An Elaborate New Linker System Significantly Enhances the Efficacy of a HER2-antibody-drug Conjugate Against Refractory HER2-positive Cancers.

Seol Hwa Shin[†], *Yun-Hee Park*[†], *Seok Soon Park, Eun Jin Ju, Jin Park, Eun Jeong Ko, Dong Jun Bae, Sang-Yeob Kim, Chul-Woong Chung, Ho Young Song, Se Jin Jang, Seong-Yun Jeong*[*], *Si Yeol Song*[*], *Eun Kyung Choi*[*]

correspondence to: syj@amc.seoul.kr, coocoori@amc.seoul.kr and ekchoi@amc.seoul.kr

This PDF file includes:
Figure S1, S2 and Video S1
Video S1 is uploaded in separate .mp4 files due to its large size.

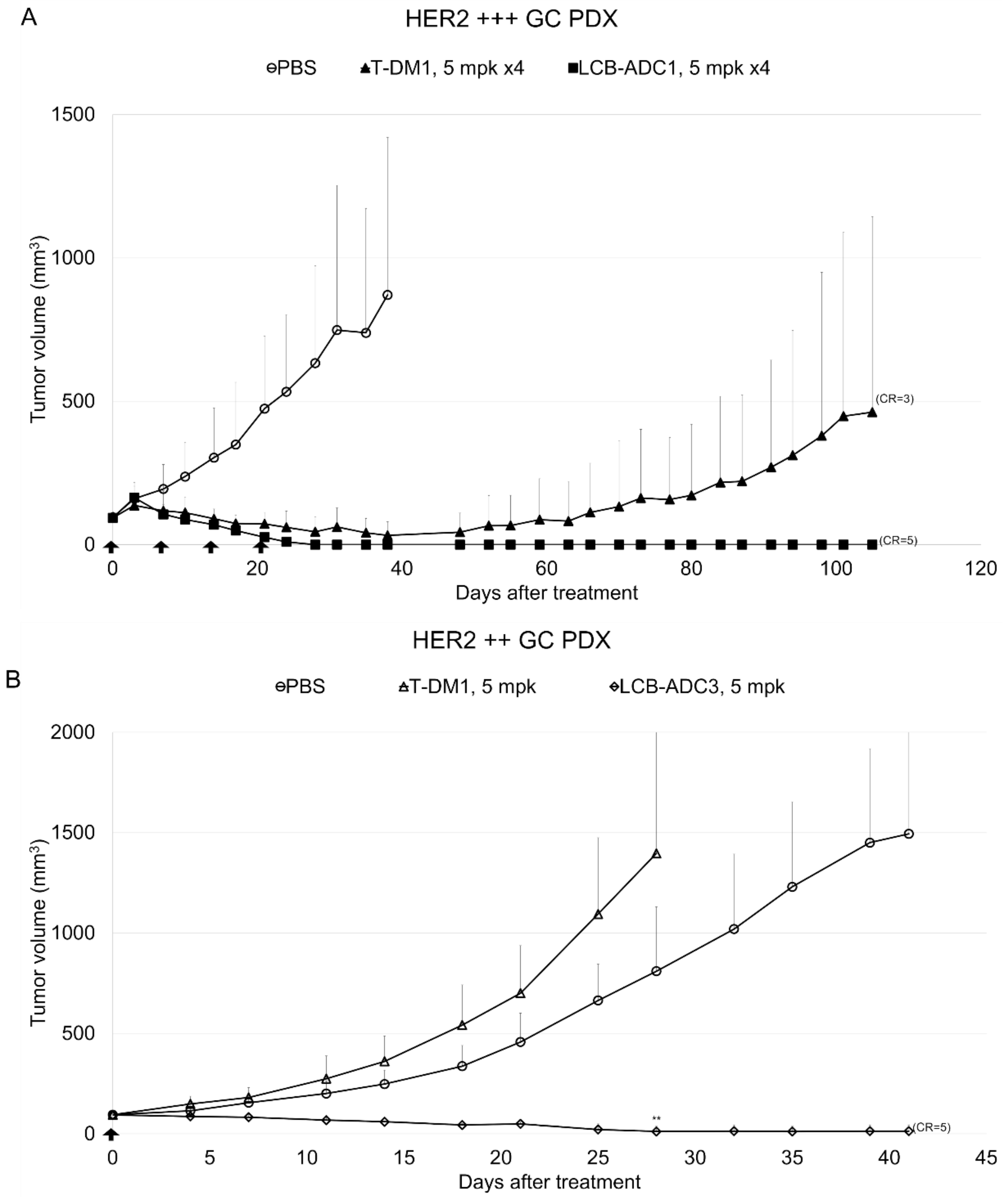

Figure S1. In vivo efficacy of LCB-ADCs and T-DM1 in the HER2-positive GC PDX model. **(a)** Mice bearing HER2 +++ GC patient-derived tumors were *i.v.* administered with LCB-ADC1 or T-DM1 at 5 mg/kg, weekly for 4 weeks (↑). Data are presented as the mean ± standard deviation. **(b)** Effect of LCB-ADC3, T-DM1 in a HER2 ++ GC PDX model. Drugs were *i.v.* injected at 5 mg/kg once (↑). Data values are presented as the mean ± standard deviation. **p<0.01 (T-DM1 vs LCB-ADC3).

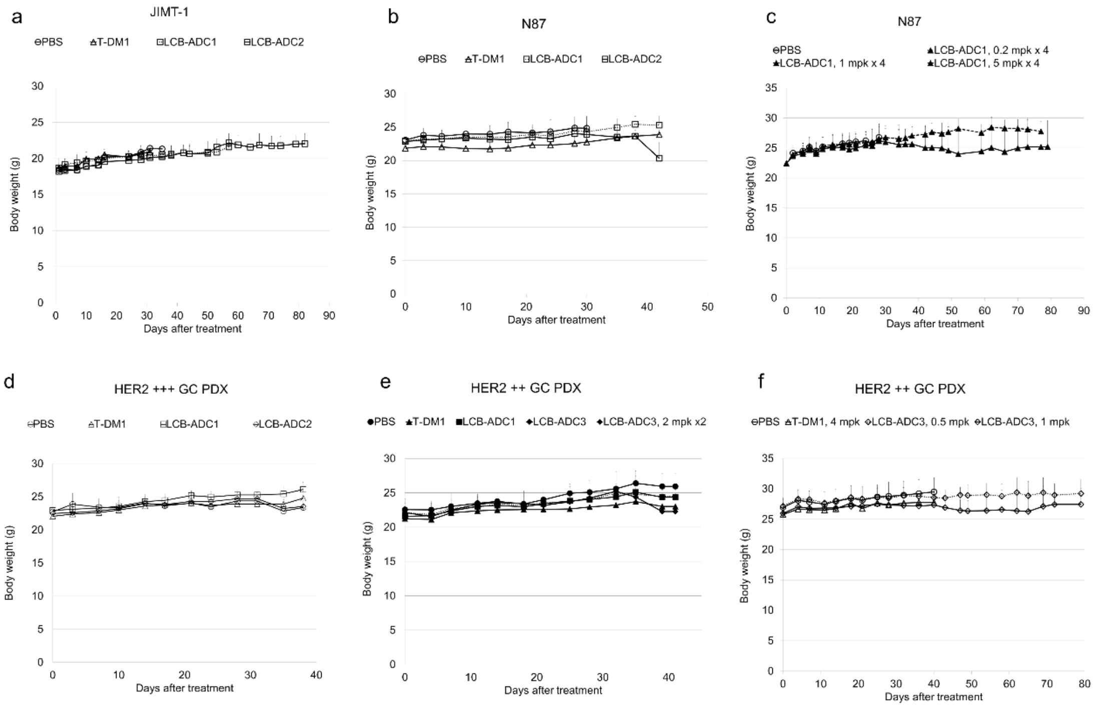

Figure S2. Body weight of mice in the HER2 positive CDX and PDX models corresponding to (a) figure 5a, (b) figure 5b, (c) figure 5d, (d) figure 6a, (e) figure 6b, and (f) figure 7.

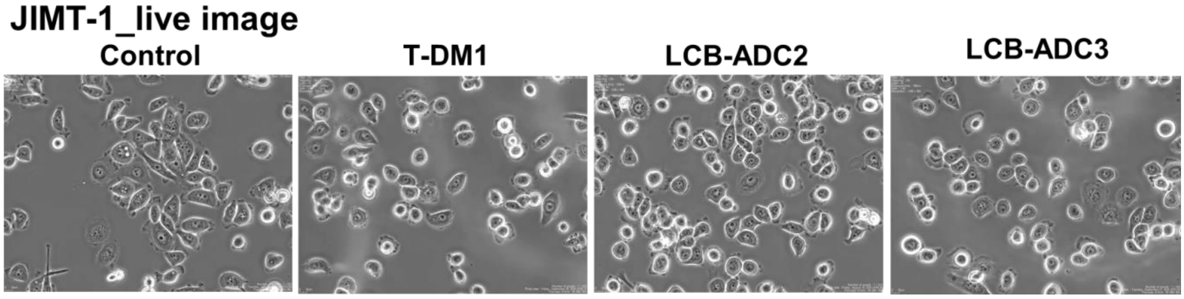

Video S1. *See the separate movie file to view the live imaging

Live imaging of JIMT-1 cells treated with the LCB-ADCs

JIMT-1 cells were treated with T-DM1, LCB-ADC2 or LCB-ADC3 at 0.25 μg/ml for 72 h, and then recorded in real-time.
